# Probiotic strain *Stenotrophomonas acidaminiphila* BJ1 degrades and reduces chlorothalonil toxicity to soil enzymes, microbial communities and plant roots

**DOI:** 10.1186/s13568-017-0530-y

**Published:** 2017-12-23

**Authors:** Qingming Zhang, Muhammad Saleem, Caixia Wang

**Affiliations:** 1 College of Plant Health and Medicine, Qingdao Agricultural University, Qingdao, 266109 Shandong China; 2 Department of Plant and Soil Sciences, University of Kentucky, Lexington, KY 40546-0312 USA

**Keywords:** Soil probiotics, Chlorothalonil, Biodegradation, Detoxification, Soil enzymes

## Abstract

Chlorothalonil, a non-systemic and broad-spectrum fungicide, is widely used to control the pathogens of agricultural plants. Although microbial degradation of chlorothalonil is known, we know little about the colonization and degradation capacity of these microbes in the natural and semi-natural soil environments. Therefore, we studied the colonization and detoxification potential of a chlorothalonil degrading *Stenotrophomonas acidaminiphila* probiotic strain BJ1 in the soil under green conditions. The results from polymerase chain reaction-denaturing gradient gel electrophoresis demonstrated that probiotic strain BJ1 successfully colonized the soil by competing with the native biota. Moreover, the bacterial inoculation stimulated some members of indigenous soil microbial communities. Meantime, the degradation half-life of chlorothalonil decreased from 9.0 to 4.9 days in the soil environment. Moreover, the results from enzymatic activities and micronucleus test of *Vicia faba* root tips showed that the probiotic strain BJ1 reduced the ecotoxicity and genotoxicity of chlorothalonil in the soil. We suggest that probiotic strains like BJ1 could potentially alleviate the toxic effects of pesticides on soil microbes and plant roots under greenhouse conditions.

## Introduction

Chlorothalonil (2,4,5,6-tetrachloro-1,3-benzenedicarbonitrile, CAS No. 1897-45-6), a non-systemic and broad-spectrum fungicide, is extensively used to control fungal pathogens of vegetables and fruits in modern agriculture (Carlo-Rojas et al. 2004; Liang et al. 2010). Correspondingly, the environmental concerns and toxic effects of chlorothalonil on soil biological properties are studied extensively (Chen et al. 2001; Hussain et al. 2009a; Teng et al. 2017). The half-life of chlorothalonil in soil ranges from several days to 1 year, depending on the soil type, applied concentration, and application frequency (Sigler and Turco 2002; Wu et al. 2012). While some studies have shown the adverse effects of chlorothalonil on soil microbial community composition, abundance, and enzymatic activities (Hussain et al. 2009b; Teng et al. 2017; Wu et al. 2012; Yu et al. 2006). Owing to a reduced volatilization, illumination intensity, and rainfall, the chlorothalonil accumulates to several folds and, thus causes toxicity to soil organisms and plant roots (Jin et al. 2014). Therefore, it is necessary to develop efficient and eco-friendly approaches to remediate chlorothalonil-contaminated soil, especially under greenhouse conditions.

Several methods such as hydrolysis, photolysis, catalytic and bio-degradation are known to reduce fungicide residues in the soil environment. (Ghauch and Tuqan 2008; Rouchaud et al. 1988; Sakkas et al. 2002; Shi et al. 2011; Wu et al. 2016). Among these, the microbial driven removal of fungicides is a cost-effective and eco-friendly way to detoxify the contaminated sites. While some microbial strains such as *Bacillus cereus* NS1, *Pseudomonas* CDS-8, and *Ochrobactrum lupini* TP-D1, etc., are known to degrade chlorothalonil in the soil and water environments (Shi et al. 2011; Wu et al. 2016; Zhang et al. 2007). The *Stenotrophomonas acidaminiphila* BJ1, a previously isolated strain in our laboratory, could remove 93.2% of chlorothalonil (50 mg L^−1^) within 7 days in the optimal liquid media. Although microbial-driven removal of chlorothalonil is known (Shi et al. 2011; Zhang et al. 2014a), there is a debate about whether these strains colonize and compete for the same ecological niche with native species in the contaminated sites. Moreover, the role of microbial species in reducing chlorothalonil toxicity to soil enzymes, microbes, and plant roots, remains to be studied in situ.

The polymerase chain reaction-denaturing gradient gel electrophoresis (PCR-DGGE) is widely used to study microbial community structure and diversity in various environments (Deng et al. 2015; Malinich et al. 2017; Muyzer and Smalla 1998; Nicol et al. 2008). While some studies also used PCR-DGGE to investigate the colonization ability of microbial strains in the soil. For example, Cunliffe and Kertesz (2006) examined the colonization ability of *Sphingobium yanoikuyae* B1 and its effect on the native bacterial community in three different soils using PCR-DGGE targeting 16S rRNA genes. Similarly, Kong et al. (2014) demonstrated the colonization of *Alcaligenes faecalis* JBW4 in endosulfan-contaminated soil using the same approach. Moreover, the micronucleus assay is successfully applied to determine the soil genotoxicity induced by pesticides and other pollutants (Jin et al. 2014; Zhang et al. 2014b). Thus, using state-of-the-art methods such as PCR-DGGE, micronucleus and soil enzyme assays, we aimed to investigate the ability of probiotic strain BJ1 to colonize and then degrade chlorothalonil in soil under greenhouse conditions. We also investigated the impact of probiotic strain BJ1 on chlorothalonil toxicity to the microbial communities, soil enzymes and *Vicia faba* root tips in the greenhouse soil. We hypothesized that the probiotic strain BJ1 would colonize successfully chlorothalonil-contaminated soil due to its potential to use the fungicide as a carbon and nutrient source. While bacterial colonization of soil may reduce the toxicity of chlorothalonil to native soil biota, enzymes, and plant roots. The results will help us understand the potential of biological resources in improving the soil health and quality for safe crop production under greenhouse environment.

## Materials and methods

### Chemicals, soil, *V. faba*, and probiotic strain BJ1

We obtained Chlorothalonil (97%) from Qingdao Hansen Biologic Science Co., Ltd., Qingdao, China. All other chemicals were of analytical grade, and the Milli-Q water was used throughout the experiment. We collected the experimental soil form a vegetable field (5–20 cm depth) located in the Agricultural Farm of Qingdao Agricultural University, China. The soil has no previous history of chlorothalonil use. The soil was a silt loam with following properties: pH, 6.32; sand, 27.3%; silt, 56.8%; clay, 15.9%; total organic carbon, 17.5 g kg^−1^ dry weight (DW); total N, 1.35 g kg^−1^ DW; total P, 1.04 g kg^−1^ DW; and cationic exchange capacity, 15.6 cmol kg^−1^. The collected soil was air-dried, sifted through a 2-mm sieve, and then stored at room temperature for 48 h prior to use. The *V. faba* seeds were purchased from a local market. The *Stenotrophomonas acidaminiphila* probiotic strain BJ1 was isolated in our laboratory and deposited in China Center for Type Culture Collection (Wuhan, China) with preservation number M2011475. We prepared inocula following the method of Kong et al. (2014). Briefly, about 30 mL of liquid Luria–Bertani medium was inoculated with a single colony of probiotic strain BJ1 in the Erlenmeyer flask (100 mL volume). The inoculum flask was incubated aerobically with continuous shaking (130 rpm) at 30 °C for 24 h in the dark. After incubation, the medium was centrifuged at 8000 rpm for 8 min at room temperature, and then the pellet was washed twice in phosphate buffer (0.05 M Na_2_HPO_4_/NaH_2_PO_4_, pH 7.0). The washed cells were diluted in the same buffer to a concentration of OD_600_ = 1.0 (approximately 3.5 × 10^8^ colony forming units (CFU) mL^−1^).

### Experimental design

We conducted experiments in brown pots (3 L volume) in a plastic-covered greenhouse located in the Agricultural Farm of Qingdao Agricultural University, China. We had two experimental treatments; (i) natural soil with chlorothalonil (NC), and (ii) natural soil with chlorothalonil and probiotic strain BJ1 (NCB). In NC treatment, the soil sample (1.5 kg DW) was spiked with chlorothalonil dissolved in sterile distilled water to give a final concentration of 20 mg kg^−1^ of dry soil. The treatment with 20 mg chlorothalonil kg^−1^ soil corresponds to the tenfolds of recommended dosage. In the NCB treatment, NC soil was inoculated with the bacterial suspension to make a final density of 10^7^ cfu g^−1^ dry soil (Zhang et al. 2014a). Same amount of soil without chlorothalonil and bacterial inoculation was used as the control. All soil treatments were in triplicate. The soil moisture contents were maintained at 60% of water holding capacity by periodic addition of sterile water. The soil pots were covered with perforated polypropylene sheets and incubated for 28 days in the greenhouse. We collected samples from each treatment at 0, 3, 7, 10, 14, 21, and 28 days of incubation to analysis the chlorothalonil in the soil. However, the soil samples taken after 7 and 14 days were also used to determine colonization and detoxification potential of probiotic strain BJ1 in the soil.

### Extraction and determination of chlorothalonil in soil

The chlorothalonil residues in soil were extracted and determined following a standard method (Wu et al. 2012). Briefly, we placed 10 g of dry soil and 40 mL *n*-hexane-dichloromethane (1:1, v/v) into a 150 mL Erlenmeyer flask. After 20 min ultrasonication, the organic layer was filtered through a Buchner funnel into a flat-bottom flask. Same procedure was repeated one time. All collected filtrates were concentrated to approximately 1 mL on a rotary evaporator at 45 °C, and further dried under a stream of nitrogen. The dried chemical mass was dissolved in 10 ml *n*-hexane for instrumental analysis. The chlorothalonil contents were determined using an Agilent 6890 N gas chromatography system equipped with an electron capture detector and an HP-5 capillary column (30 m × 0.32 mm × 0.25 μm). During analysis, the injector and detector temperatures were 250 and 300 °C, respectively. The oven temperature was programmed as follows: 80 °C for 1 min, increasing to 260 °C at 25 °C min^−1^, and holding for 4 min. Nitrogen was used as carrier gas at a constant flow of 2 mL min^−1^. The injection volume was 1 μL using the splitless injection mode.

### DGGE experiment

The soil total DNA was extracted using PowerSoil^®^ DNA Isolation Kits (MO BIO Laboratories, Carlsbad, CA. USA) following manufacturer’s instructions. The extracted DNA was quantified using a spectrophotometer (Biophotomether, Eppendorf, Germany). The normalized DNA samples were used as templated for PCR amplification. The primers GC-338F (5′-CGCCCGCCGCGCGCGGCGGGCGGGG CGGGGGCACGGGGGGCCTACGGGAGGCAGCAG-3′) and 518R (5′-ATTACCGCGGCTGCTGG-3′) were used to amplify the V3 region of the 16S rDNA gene (Muyzer et al. 1993). The PCR amplification reaction mixture contained 10×PCR buffer 5 μL, dNTP (2.5 mM) 3.2 μL, rTaq (5 U μL^−1^) 0.4 μL, each primer (20 μM) 1 μL, DNA template 1 μL (50 ng), and sterile filtered milli-Q water to volume of 50 μL. The PCR was conducted in Biometra T-Gradient Thermocycler (Biometra, Goettingen, Germany). The thermal program was set as follows: initial denaturation at 94 °C for 5 min, followed by 30 cycles of denaturation at 94 °C for 1 min, annealing at 55 °C for 45 s, and extension at 72 °C for 1 min. Finally, the extension was performed at 72 °C for 10 min. The PCR products were purified using the DNA Gel Extraction Kit (Omega, Germany) following the manufacturer’s instructions.

The DGGE analysis was performed in a D-code Universal Mutation Detection System (Bio-Rad, Hercules, USA) in a 7% polyacrylamide gel containing a linear 35–55% denaturant gradient with a constant voltage of 150 V at 60 °C for 5 h. The gels were then stained with silver nitrate, photographed, and analyzed using Quantity One software (Bio-Rad, USA). The Shannon–Wiener index, evenness, and richness were used to compare the bacterial community diversity between the samples. The selected bands were excised from the polyacrylamide gel, and the amplified DNA was recovered using Poly-Gel DNA Extraction Kit (OMEGA Bio-Tek, USA). The recovered DNA products were used as template for reamplification of 16S rDNA gene using 338F and 518R primers.

### Soil bacterial population and enzymatic activity

We used plate counting method to estimate soil heterotrophic bacterial population. Briefly, a 10 g of dry soil was placed into 250 mL Erlenmeyer flask containing 90 mL of sterile water. The flasks were shaken at 250 rpm for 20 min, and then stationed for 5 min. We plated suitable dilutions of soil suspension (100 μL) on the beef-extract-peptone-agar medium in a 9 cm Petri dish, and then these were incubated at 30 °C for 2 days until colonies appeared.

The soil urease activity was measured using the method of Liu et al. (2014) whereas the results are expressed as μg NH_4_^+^ g^−1^ soil h^−1^. The dehydrogenase activity was determined according to the method of Tabatabai (1994), and the results are expressed as μg triphenyl formazan (TPF) g^−1^ soil h^−1^. Moreover, the invertase activity was measured following the procedure of Hu et al. (2006), and the results were expressed as mg glucose g^−1^ soil h^−1^.

### Micronucleus test

The micronucleus test was performed following the protocol of Jin et al. (2014). Each treatment contained six slides. We counted one thousand cells per root tip using optical microscope and then to record the number of micronuclei. The micronucleus (MCN) permillage and pollution index (PI) were calculated by the equations: MCN ‰ = number of cells containing micronucleus/total number of cells counted; PI = MCN ‰ of sample/MCN ‰ of the control. The extent of pollution was according to the following criteria: PI < 1.5, zero pollution, 1.5–2.0, slight pollution; 2.0–3.5, intermediate pollution; and > 3.5, heavy pollution (Kong et al. 1998).

### Statistical analysis

Statistical analyses were carried out using SPSS 18.0 software (SPSS, Chicago, USA), and all data are presented as mean ± standard deviation (SD). Analysis of variance was used to test all data. The Dunnett t test was employed to assess the differences between treatments at a confidence level of 0.05. All the figures in this study were generated using Origin 8.6 software (OriginLab, USA).

## Results

### Validation of residue analysis method and degradation of chlorothalonil in soil

**Fig. 1:**
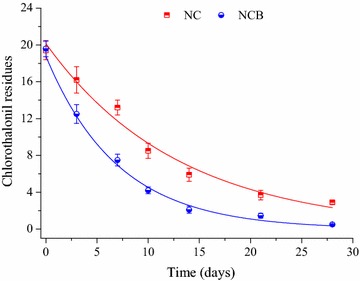
Degradation of chlorothalonil in the soil after different treatments. All values are mean ± SD of three replicates

The method of chlorothalonil residue analysis was validated by recovery experiment. The recoveries of chlorothalonil (0.2, 2, and 20 mg kg^−1^) from soil ranged from 79.7–92.4%, with relative standard deviation ranging from 3.2 to 5.5% (Table 1), which indicated that the extraction methods were efficient for chlorothalonil residue analysis. As shown in Fig. 1, the dissipation kinetics of chlorothalonil (20 mg kg^−1^) followed the first-order decay model in the treatments of NC and NCB. The kinetics formulas were y = 20.15e^−0.0771x^, r^2^ = 0.96, and y = 18.78e^−0.1422x^, r^2^ = 0.95 for NC and NCB treatments, respectively. The calculated half-lives of chlorothalonil in NC and NCB were 9.0 and 4.9 days, respectively, indicating that the degradation rate of chlorothalonil in soil mixed with the probiotic strain BJ1 (NCB) was obvious faster than that in the soil without the probiotic strain BJ1 (NC).

**Table 1:** Recoveries of chlorothalonil in soil

Concentration (mg/kg)	Recovery (%)			Average recovery (%)	RSD (%)
	1	2	3		
0.2	82.4	88.6	79.7	83.6	5.5
2	86.5	90.3	84.8	87.2	3.2
20	92.4	89.3	85.6	89.1	3.8

*RSD* relative standard deviation

### DGGE analysis

**Fig. 2:**
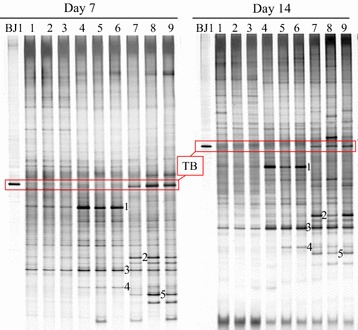
DGGE profile of strain BJ1 and soil bacteria. Lanes: 1, 2, and 3, Control; 4, 5, and 6, NC treatment; 7, 8, and 9, NCB treatment. TB: target band

In this study, the probiotic strain BJ1 whether could colonize the chlorothalonil-contaminated soil and alter the indigenous communities were investigated using DGGE experiment. As shown in Fig. 2, the target band was detected in the NCB soil on day 7 and 14, suggesting that probiotic strain BJ1 successfully colonized the chlorothalonil-contaminated soil. However, the intensity of target band at 7 days was weaker than that at 14 days. Compared to the control, the intensities of some bands such as band 1, 3, and 4 were obvious increased in treated chlorothalonil soil (NC treatment). The DNA sequences of bands 1 (GenBank accession number, MF806579), 3 (MF806581), and 4 (MF806582) matched (97–98% similarity) with the sequences of bacterial species such as *Pseudomonas pseudoalcaligenes*, *Pseudomonas glareae*, and *Rhizobacter gummiphilus*, respectively. In the NCB treatment, interestingly, the band 1 disappeared whereas the intensities of bands 2 (MF806580, 97% sequence similarity with *Lysobacter yangpyeongensis*) and 5 (MF806583, 100% sequence similarity with *Bartonella apis*) increased continuously (Fig. 2).

**Fig. 3:**
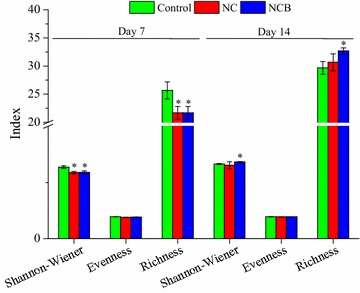
Diversity index of bacterial community in soil with different treatments. Each column represents the mean of three replicates, and the error bars represent the standard deviations. Asterisk (*) indicates statistical significance of treatments in comparison to the control at *p* < 0.05

The changes in bacterial community composition in the chlorothalonil-contaminated soil alone or together with probiotic strain BJ1 at various intervals were presented in Fig. 3. On day 7, the Shannon–Wiener index and richness significantly decreased in the NC and NCB than control treatment (Fig. 2). However, with time, the Shannon–Wiener index and richness values recovered to the control level in the NC treatment at 14 days. Interestingly, Shannon–Wiener index and richness values increased in NCB than control treatment.

### Soil bacterial population and enzymatic activity

As shown in Table 2, the culturable bacterial population was significantly (*p* < 0.05) decreased in chlorothalonil treatment (NC) at 7 and 14 days, by 41.6 and 21.7%, respectively. However, in presence of probiotic strain BJ1 (NCB), the bacterial population increased and recovered to the control level on day 14. Similar to the bacterial population, during the whole experimental period, the activities of three soil enzymes (urease, dehydrogenase, and invertase) were all significantly inhibited by chlorothalonil treatment (NC). In the NCB treatment, the inhibition effect of chlorothalonil on the activities of three soil enzymes was reduced compared to the NC treatment. And their activities were all recovered to the control levels (Table 2).

**Table 2:** Bacterial population and enzymatic activity in the soil treated with chlorothalonil or/and strain BJ1

Time	Treatment	Bacteria	Urease	Dehydrogenase	Invertase
7 days	Control	43.82 ± 4.34 a	37.25 ± 4.72 a	34.30 ± 3.84 a	0.64 ± 0.07 a
	NC	25.60 ± 5.25 c	18.63 ± 3.25 c	20.45 ± 3.20 c	0.35 ± 0.05 b
	NCB	34.32 ± 4.27 b	27.32 ± 3.46 b	25.46 ± 4.02 b	0.58 ± 0.06 a
14 days	Control	42.22 ± 6.43 a	40.22 ± 4.63 a	33.64 ± 3. 63 a	0.72 ± 0.07 a
	NC	33.45 ± 5.40 b	28.40 ± 4.38 b	26.44 ± 3.04 b	0.54 ± 0.05 b
	NCB	38.18 ± 3.85 ab	37.40 ± 5.24 a	31.70 ± 4.25 ab	0.68 ± 0.08 a

Bacterial population (10^6^ g^−1^ soil), Urease activity (μg NH_4_^+^ g^−1^ soil h^−1^), Dehydrogenase activity (μg TPF g^−1^ soil h^−1^). Invertase activity (mg glucose g^−1^ soil h^−1^). All values are mean ± SD of triplicate samples. Different letters denote significant differences at the 0.05 confidence level between treatments at the same exposure time

### Micronucleus assay of *V. faba* root tip

**Fig. 4:**
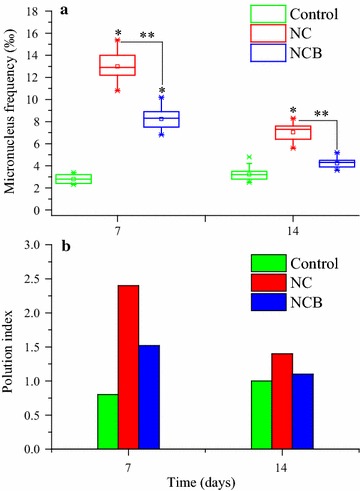
Micronucleus frequency (**a**) and pollution index (**b**) of *Vicia faba* treated with different soil solutions. Asterisk (*) indicates statistical significance of treatments in comparison to the control at *p* < 0.05. Two asterisks (**) indicates statistical significance between NC and NCB at *p* < 0.05

The genotoxicity of soil contaminated with chlorothalonil was evaluated by micronucleus assay of *V. faba* root tip. As shown in Fig. 4a, the micronucleus frequency of *V. faba* root tips under chlorothalonil exposure was significantly higher (*p* < 0.05) than that of the control at 7 and 14 days. In the presence of probiotic strain BJ1 (NCB), the micronucleus frequency of *V. faba* root tips was significantly lower (*p* < 0.05) than that of NC treatment, and it recovered to the control level on day 14. According to the pollution index (Fig. 4b), the chlorothalonil (20 mg kg^−1^) caused intermediate pollution in a short interval of time (7 days) while probiotic strain BJ1 decreased the level of chlorothalonil pollution to almost zero level till the end of experimental period.

## Discussion

It is well known that microbial degradation is a cost-effective and eco-friendly way to remediating contaminated sites. In this study, a probiotic strain BJ1 was used to remove chlorothalonil in soil under greenhouse conditions. In presence of probiotic strain BJ1, the half-life of chlorothalonil was changed from 9.0 to 4.9 days, indicating that bacterial inoculation increased the rates of chlorothalonil degradation in the soil. The results were in lined with previous studies which reported chlorothalonil can be effectively degraded by addition of exogenous degrading bacteria to soil environment (Regitano et al. 2001; Yu et al. 2006; Zhang et al. 2014a). Moreover, our results also advocate the notion that microbial-driven bioremediation is an effect way to clean the contaminated sites (Gharasoo et al. 2015; Hussain et al. 2009b).

In PCR-DGGE experiment, we found the target band in the NCB soil on the 7th and 14th days (Fig. 2), indicating that probiotic strain BJ1 successfully colonized the chlorothalonil-contaminated soil. Over time, however, the intensity of target band gradually decreased, thus suggesting a decreased growth of the degrader strain. This could be due to either a significant decrease in pesticide residues that act as nutrient source for probiotic strain BJ1 in the soil (Hussain et al. 2009a, Hussain et al. 2009b) and/or a resurgence of native microbial species under reduced anthropogenic pressure to reoccupy the ecological niches (Kong et al. 2014; Saleem et al. 2015; Weber et al. 2001). But nevertheless, the presence of probiotic strain BJ1 in the soil at 14 days suggested that introduced strain has successfully colonized soil; however, its niche breadth depended on the contents and bioavailability of resources (pesticide residues). Given that chlorothalonil could affect bacterial communities, we observed changes in the intensities of DGGE bands. Notably, the band 1, 3 and 4 in treated chlorothalonil than control soil increased in their intensity. In NCB treatment only, interestingly, the band 1 disappeared whereas the intensities of bands 2 and 5 increased continuously (Fig. 2). The increase in their intensity, in general, indicated that some indigenous microbes, in addition to probiotic strain BJ1, were either able to utilize chlorothalonil and/or its metabolic by-products as source of energy and nutrients to multiply (Hussain et al. 2009a, Hussain et al. 2009b; Saleem and Moe 2014; Yu et al. 2006). The later argument seems to be more relevant because the degradation rate of chlorothalonil did not increase significantly during this time. Similar effects of other pesticides such as imidacloprid and iprodione on soil bacterial populations are reported in previous studies (Cycoń et al. 2013; Wang et al. 2004). Moreover, fungicide treatment (NC) significantly (*p* < 0.05) reduced culturable bacterial population (Table 2). This observation was agreed with previous studies in which application of higher concentration of chlorothalonil had an inhibition effect on soil bacteria (Kumar Singh et al. 2002; Yu et al. 2006). To some extent, DGGE data corresponded to culturable bacterial abundance; though lower than control on day 7, it recovered to control level on day 14. This further indicated that probiotic strain BJ1 successfully colonized the soil and decreased the inhibition effect of chlorothalonil by accelerating its degradation. It is very likely that probiotic strain BJ1 might had influenced the microbial community composition and survival of indigenous microorganisms by reducing the anthropogenic pressure in the soil. An increase in the intensity of band 2 and 5 in the presence of probiotic strain BJ1 nevertheless predicts a synergistic relationship with indigenous microbes and probiotic bacteria. This may also suggest that probiotic strain BJ1 stimulated these microbes by producing some public goods. There are few evidence that support our results. For instance, an endosulfan-degrading *Alcaligenes faecalis* strain JBW4 showed a synergistic relationship with some indigenous microorganisms (Kong et al. 2014). Moreover, we also observed changes in bacterial community composition in the chlorothalonil-contaminated soil alone or together with probiotic strain BJ1 at various intervals. Although community evenness did not differ, the Shannon–Wiener index and richness significantly decreased in the NC and NCB than control treatment at 7 days (Fig. 3). However, with time, these values recovered to the control level in the NC treatment at 14 days. Interestingly, compared to the control, the Shannon–Wiener index and richness values increased significantly (*p* < 0.05) in the NCB treatment. These results suggest that chlorothalonil inhibited the soil bacterial communities in a short time whereas indigenous microbiome recovered faster in the presence of probiotic strain BJ1, due to a greater resilience (Kong et al. 2014). However, the absence of few bands in the NCB treatment suggests the inhibitory effect of probiotic strain BJ1 on indigenous microbes in the presence of chlorothalonil (Bending et al. 2007; Sigler and Turco 2002). Overall, a positive impact of probiotic strain BJ1 on soil microbial communities helped in reducing the half-life of chlorothalonil in the soil.

Next to this, we also determined the toxicity of chlorothalonil to soil enzymes including urease, dehydrogenase, and invertase. These enzymes play important roles in the cycling of organic matter and nitrogen in the soil environment (Burns 1982; Hussain et al. 2009a). Both fungicide treatments (NC and NCB) inhibited enzyme activities at 7 days (Table 2), thus indicating the toxicity of chlorothalonil to soil enzymes for a short time interval even in the presence of probiotic strain BJ1. However, at 14 days, the activities of all enzymes in the NCB than NC treatment recovered to the control levels (Table 2) that nevertheless suggest the potential of probiotic strain BJ1 and indigenous microbial communities in reducing the adverse effect of chlorothalonil on soil enzymatic activities (Kong et al. 2014; Yu et al. 2006). While the variations in soil activities coincided with the changes in bacterial population under chlorothalonil exposure with and/or without BJ1.

Finally, we investigated the genotoxicity of chlorothalonil using micronucleus assay of *V. faba* root tip (Fig. 4). The micronucleus frequency of *V. faba* root tips under chlorothalonil exposure was significantly higher (*p* < 0.05) than that of the control at 7 and 14 days. These results are partly similar with that of Jin et al. (2014) who also observed an increase in the micronucleus frequency of *V. faba* root tips treated with the solution of soil contaminated with chlorothalonil under greenhouse condition. Therefore, we further confirmed that chlorothalonil could cause genotoxicity to the root cells. The micronucleus frequency of *V. faba* root tips in NCB than NC was significantly lower (*p* < 0.05), and it recovered to the control level. This indicated that addition of probiotic strain BJ1 could significantly reduce (*p* < 0.05) the genotoxicity of chlorothalonil to plant roots.

In summary, the results of this study showed that the probiotic strain BJ1 successfully colonized the chlorothalonil-contaminated soil. Moreover, it metabolized the chlorothalonil while stimulated the indigenous microbial communities that further reduced the toxicity of fungicide to soil enzymes and plant roots. We conclude that the probiotic strain BJ1 has the potential to detoxify the chlorothalonil and stimulate indigenous microbial communities for higher soil health and function.

## Abbreviations

PCR-DGGE: polymerase chain reaction-denaturing gradient gel electrophoresis
DW: dry weight
CFU: colony forming units
NC: natural soil with chlorothalonil
NCB: natural soil with chlorothalonil and probiotic strain BJ1
TPF: triphenyl formazan
MCN: micronucleus
PI: pollution index
SD: standard deviation
